# Temperature models of development for *Necrodes littoralis* L. (Coleoptera: Silphidae), a carrion beetle of forensic importance in the Palearctic region

**DOI:** 10.1038/s41598-022-13901-y

**Published:** 2022-06-11

**Authors:** Joanna Gruszka, Szymon Matuszewski

**Affiliations:** 1 Laboratory of Criminalistics, Adam Mickiewicz University, Św. Marcin 90, 61-809 Poznań, Poland; 2 Center for Advanced Technologies, Adam Mickiewicz University, Uniwersytetu Poznańskiego 10, 61-614 Poznań, Poland; 3 Department of Animal Taxonomy and Ecology, Adam Mickiewicz University, Uniwersytetu Poznańskiego 6, 61-614 Poznań, Poland

**Keywords:** Entomology, Ecology

## Abstract

Analysis of insects can provide evidence in death cases, for example, by answering the question about the time of death. Apart from flies, beetles are the second most useful insect group in forensic entomology. To elucidate the time of death based on insect evidence, developmental models of a given species are necessary. In this study, we developed such models for *Necrodes littoralis,* a necrophagous beetle, which is common in the Palearctic region and has great potential in forensic entomology. We monitored the development at 10 constant temperatures (14–30 °C). Larvae were reared in aggregations. Thermal summation models, isomorphen and isomegalen diagrams and growth curves were derived using the data. Depending on the temperature, development lasted between about 23 and 89 days. Mortality was high at the extremes of the temperature range. The thermal summation constant for the total development was 434.7 ± 28.86 accumulated degree-days above a developmental threshold of 9.04 ± 0.55 °C. This is the first comprehensive dataset on the development of *N. littoralis*. Implications for its use in forensic casework are discussed.

## Introduction

Forensic entomology uses insects and other arthropods as evidence in legal investigations. Arthropods are used mainly in homicide, suicide and mysterious death cases, when insect evidence allows to establish some circumstances of death^1,2^. For instance, due to the ecological specialization of specific insect taxa, it is possible to determine the environment where death occurred and to answer the question whether the body was relocated after death^3,4^. Most often, however, insects are used to elucidate the time of death, usually through the estimation of minimum postmortem interval (PMI_min_)^5^. Two main methods can be used for this purpose. The first one uses the regularity of insect succession on carcasses^6,7^. Using models of insect succession on carrion for given environmental conditions and a set of insect taxa found on the death scene, we can try to estimate how much time has passed since death^8^. The second method uses the regularity of insect development, in particular a close relationship between temperature and the rate of development^1,9^. Developmental studies of necrophagous insects allow for the creation of developmental models that can be used to estimate PMI_min_.

Forensic entomologists created various kinds of developmental models. Some of them, such as the isomegalen diagram or growth curve, represent the relationship between development time and size of the larvae at a given temperature. The isomorphen diagram shows the duration of developmental stages at given temperatures. The thermal summation model (TSM) assumes that within the certain temperature range, there is a linear relationship between the rate of development and temperature and that development stops below a certain temperature^10^. Accordingly, there is some constant amount of heat that needs to be accumulated by insects of a given species to reach certain developmental landmark (e.g. hatching, pupation or eclosion). This value is called a thermal summation constant (*K*) and is expressed in accumulated degree hours (ADH) or accumulated degree days (ADD). The temperature below which the insect species ceases to develop is termed a lower developmental threshold (*D*_*0*_) and is expressed in temperature units^10^. One of the most frequently used methods to derive TSM, is the one proposed by Ikemoto and Takai^11^. It is a modification of the classic thermal summation method. Authors proposed an equation:

$$\left(DT\right)=K+{D}_{0}*D$$

where *D* is the duration of development, *D*_*0*_ is the lower developmental threshold, *K* is the thermal summation constant and *T* is the environmental temperature. The authors also suggested using the Reduced Major Axis (RMA) regression to derive TSM. Slope of the RMA model is the lower developmental threshold (*D*_*0*_) and the *y* intercept is the thermal summation constant (*K*). Although TSMs simplify complexity of insect development and ignore its substantial intraspecific variation (e.g. variation in development time between the sexes^12,13^ or between insects of different sizes^14,15^), their use enables satisfactorily accurate estimation of insect age and eventually PMI_min_^8,16,17^.

Flies are the most frequently used group of insects in forensic entomology. They usually appear on cadavers as first colonizers and age of their immature stages allow to estimate the PMI_min_ that is close to the true PMI^18^. Typically, the first colonizers are blow flies (Calliphoridae). They appear on the corpse within the first hours or even minutes after death^10,19^. During feeding, their larvae form large aggregations^20–22^. Beetles appear later on carcasses, and are often present there until the remains stage. Hence, they can be an important tool in estimating the PMI in the advanced stages of cadaver decomposition^23^. Numerous studies of insect succession on carrion and descriptions of specific forensic cases demonstrated that beetles may be useful for the estimation of PMI^6,17,24–34^. Among the forensically important families of beetles, the most frequently mentioned ones are carrion beetles (Silphidae), rove beetles (Staphylinidae), checkered beetles (Cleridae), skin beetles (Dermestidae), clown beetles (Histeridae) and sap beetles (Nitidulidae). The greatest limitation for the use of beetles in forensic entomology is the lack of developmental models.

Developmental models for only 15 species of forensically important beetles have been published so far. First models were created for *Thanatophilus micans* (Silphidae), population from South Africa^35^. The other carrion beetles with published models comprise: *Oxelytrum discicolle* population from northern part of South America^36^, *Thanatophilus mutilatus* population from South Africa^37^, *Necrodes littoralis*^15^ population from Central Europe, *Necrophila (Calosilpha) brunnicollis* population from East Asia^38^ and populations from Central Europe of *Thanatophilus sinuatus*^39^ and *Thanatophilus rugosus*^40^. Models were created also for four species of *Dermestidae*: populations from south-western Europe of *Dermestes frischi, D. undulatus* and *D. maculatus* and Chinese population of *D. tessellatocollis*^41,42^. Developmental models were also published for central European population of *Sciodrepoides watsoni* (Leiodidae: Cholevinae)^43^, Chinese^44^ and central European^45^ populations of *Creophilus maxillosus* (Staphylinidae) and Chinese populations of *Necrobia rufipes* (Cleridae)^46^ and *Omosita colon* (Nitidulidae)^47^. Although there are beetle taxa that were extensively studied (e.g. *Thanatophilus* or *Creophilus*), still many forensically useful beetle species lack developmental models.

*Necrodes littoralis* (Silphidae) is widely distributed in the Palearctic region. It prefers open and forest natural habitats, but has also been recorded in urban open and quasi-indoor habitats^24,31,34,48,49^. Larvae feed on carrion mainly in spring and summer, adult beetles have also been recorded in the fall^24,48^. The species is associated with cadavers at active and advanced decay^7,31,48,50^. *N. littoralis* prefers large carrion, on which its larvae frequently form aggregations that allow them to drive active decay similarly to blow flies^50–52^. *N. littoralis* has been reported from many forensic cases, however it has infrequently been used to estimate PMI^17,31,34,49,53–55^. Comprehensive data on its occurrence on human cadavers in France was provided by Charabidze et al.^31^. According to their analysis, *N. littoralis* (larvae or adult beetles) were present in 154 cases (1 in 8 cases examined), with 91.6% of outdoor cases (mainly forests and bushes). Most of them occurred during spring or summer. In more than 85% of the cases *N. littoralis* was observed from the early to advanced decomposition stages. Moreover, the pre-appearance interval (PAI) of adult and larval *N. littoralis* was found to be strongly related to the preceding ambient temperature^56^ and for this reason, it may easily be estimated using temperature methods for PAI^57^. Furthermore, the size of adult *N. littoralis* was found to be negatively correlated with its physiological age at maturity. Thus, such features as length or weight of adult *N. littoralis* can be useful to calibrate developmental constants for this species and to improve the accuracy of age estimation^15^. Some partial developmental datasets for *N. littoralis* were already published^15,58^. However, there is no comprehensive dataset providing all development models and based on the full temperature range for this species. This deficiency partially explains low frequency of *N. littoralis* use for the estimation of PMI. Current study aims to create the first comprehensive set of robust development models for *N. littoralis.* Some of the data used in this work was also used for previously published analyzes^15^.

## Results

### Life cycle

Adult *Necrodes littoralis* is mainly active after dark. Mating occurs usually during the night. The female lays eggs in the soil, in batches, usually 50–70 eggs in each. The first instar larvae hatch creamy white and migrate quickly in search for food. It is the period when they are very fragile and particularly vulnerable to injuries. They darken, and their cuticle hardens with time. Likewise, the second and third instar larvae are white and non-sclerotized shortly after ecdysis. When feeding is complete, third instar larvae burrow into the ground. Then they form pupal chambers by thrashing the abdomen and thus compacting the soil around them. They go through the prepupal, pupal and teneral adult stages inside the chambers. The pupa is creamy white at the beginning and with time it gradually sclerotizes and darkens. The metamorphosis ends with the appearance of tenerals—creamy white and non-sclerotized adult beetles. Over time, their cuticle hardens and darkens, giving the insects their final black color. The beetles dig out of the pupal chamber after they became fully sclerotized and colored.

### Influence of in vivo measurements

**Figure 1:**
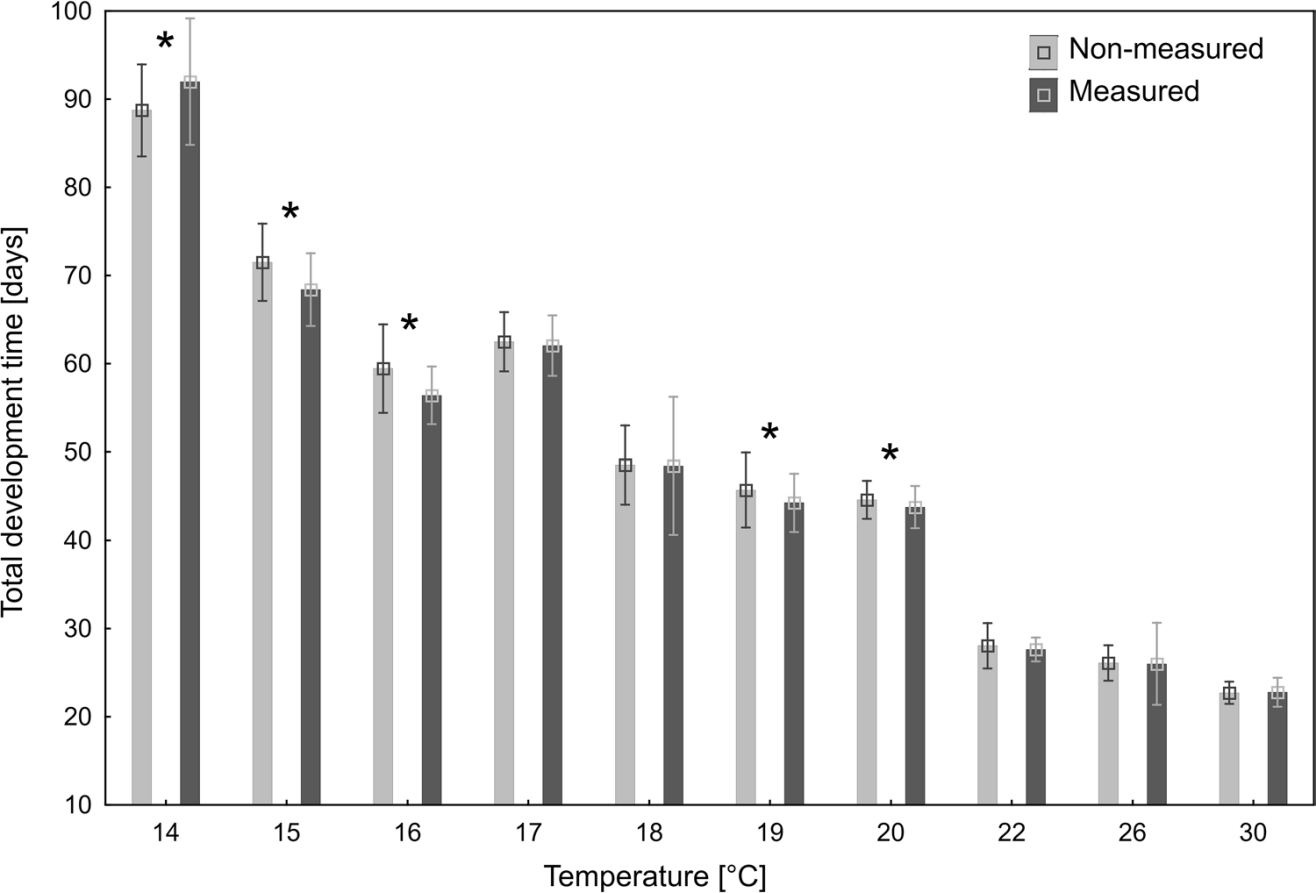
Differences in duration of total immature development between measured and non-measured specimens of *N. littoralis* at ten constant temperatures. *Statistically significant difference in Mann–Whitney *U* test at α = 0.001.

In general, non-measured beetles developed longer (Fig. 1), with significant differences recorded at 15 °C (Mann–Whitney *U* test: *Z* = 6.02, *p* < 0.001), 16 °C (Mann–Whitney *U* test: *Z* = 5.92, *p* < 0.001), 19 °C (Mann–Whitney *U* test: *Z* = 3.56, *p* < 0.001) and 20 °C (Mann–Whitney *U* test: *Z* = 3.20, *p* = 0.001). The development time of non-measured beetles was shorter only at 14 °C (Mann–Whitney *U* test: *Z* = − 3.16, *p* = 0.001). The largest difference was observed in 16 °C, where non-measured beetles developed 5.39% longer than measured beetles. However, in higher temperatures, starting from 17 °C, the differences between measured and non-measured beetles were very small (Fig. 1).

**Figure 2:**
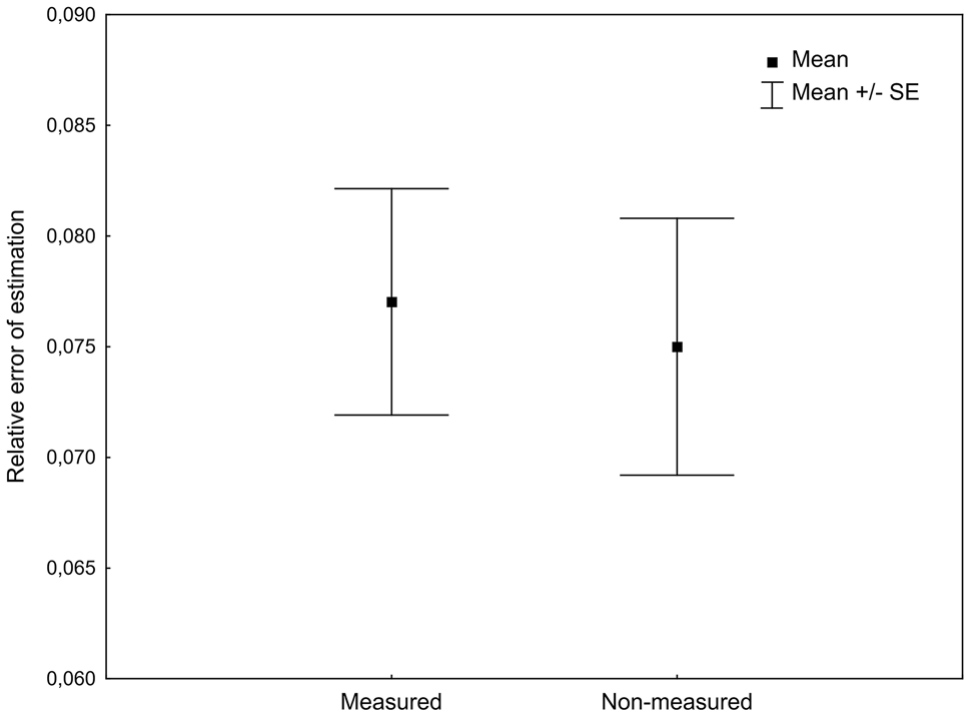
Relative error of age estimation using models calculated with measured or non-measured beetles.

Developmental models calculated using measured and non-measured specimens were only slightly different (Table 1). There were, however no significant differences in the relative error of age estimation between the tested models (Wilcoxon signed-rank test: *Z* = 0.96, *p* = 0.34, *N* = 110). Both models yielded estimates of age with the average relative error below 0.08 (Fig. 2).

**Table 1:** Thermal summation models for the total immature development of *N. littoralis*, calculated using measured or non-measured specimens.

Model	Temperature range [°C]	Thermal summation constant—*K* (SE) [degree-days]	Developmental threshold—*D*_*0*_ (SE) [°C]	r^2^	N	p
Measured	14–30	421.09 (29.06)	9.251 (0.552)	0.968	9	< 0.001
Non-measured	14–30	436.90 (29.05)	9.051 (0.548)	0.967	9	< 0.001

### Mortality and development

**Figure 3:**
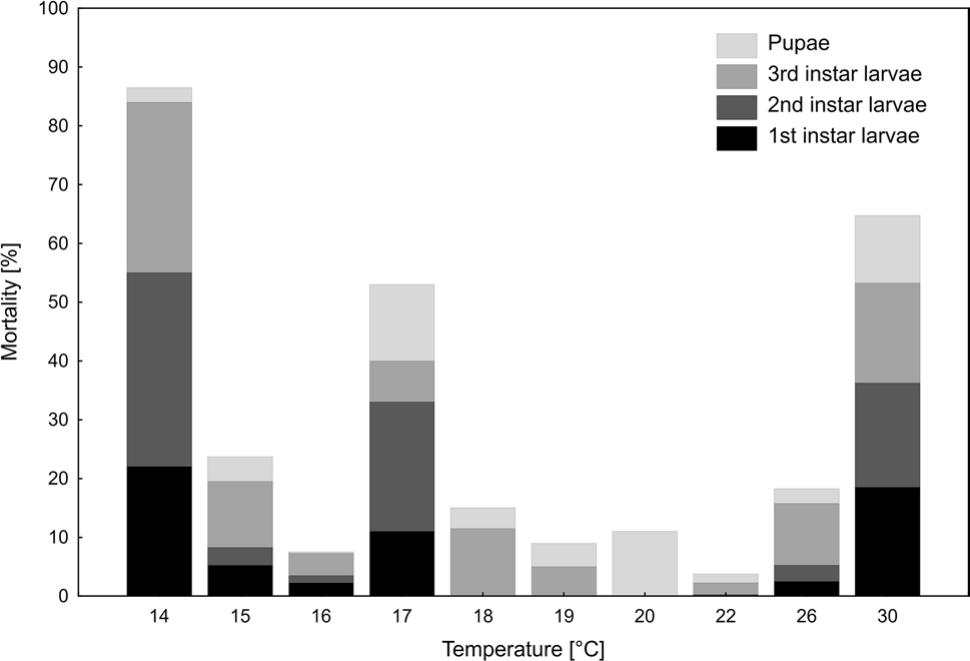
Mortality of *N. littoralis* at immature developmental stages in different rearing temperatures.

The highest mortality was observed at extreme temperatures: 86.5% at 14 °C and 64.75% at 30 °C. At the other temperatures mortality was below 25%, except for 17 °C where it was 53% (Fig. 3). There were differences between the temperatures in the distribution of deaths across the life stages (Fig. 3.) At the lowest and the highest temperatures deaths occured during all developmental stages, while at the optimal temperatures beetles died mostly at postfeeding larval or pupal stages. At 17 °C deaths were also distributed across all developmental stages. The hypothesis as to why and consequences will be presented in the discussion section.

**Figure 4:**
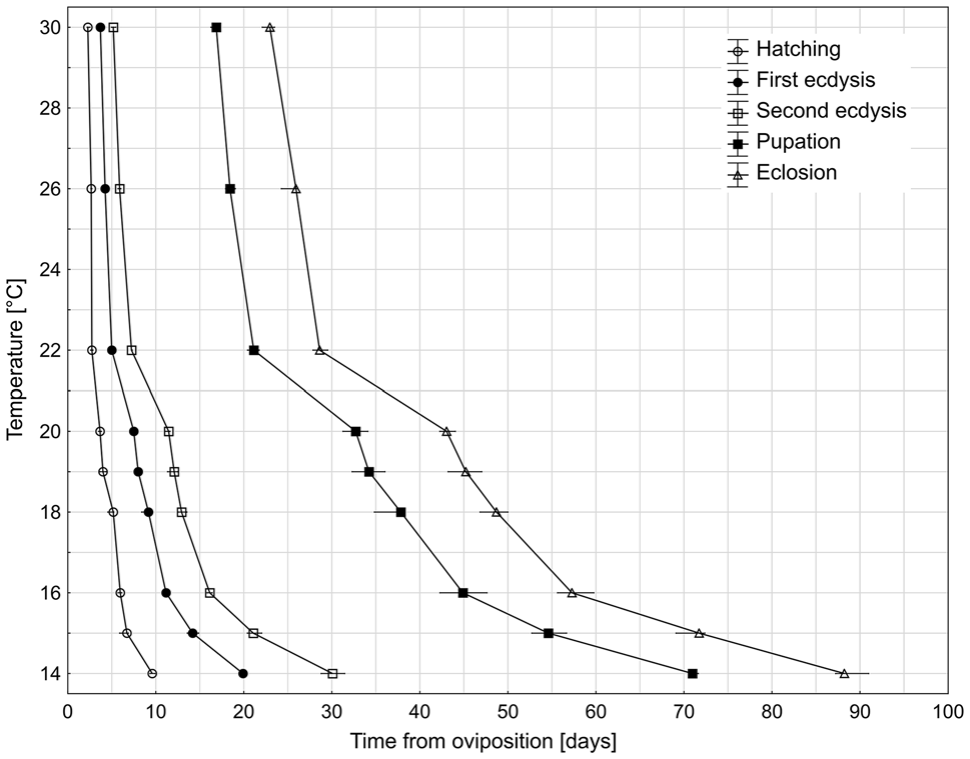
Isomorphen diagram for *N. littoralis* based on median times to reach developmental events at each of the nine temperatures tested (Supplementary Table 1). Horizontal bars represent interquartile ranges. Areas between lines represent developmental stages. Different symbols represent developmental events.

The development from egg to adult stage took between 22.84 days at 30 °C and 89.11 days at 14 °C (Table 2, Fig. 4.). Results from 17 °C deviated from the general pattern of decrease in development time with an increase in temperature. The first and second instar larvae at 17 °C developed much longer than larvae at 16 °C and 15 °C, and thus the total development time at 17 °C lengthened on average to 60.98 days. Due to this inconsistency and the exceptionally high mortality of larvae at 17 °C, we decided not to use the data from this temperature when developing the models (except for the model for hatching).

**Table. 2.** Duration of immature developmental stages of *N. littoralis* at ten constant temperatures.

Temp. [°C]	Mean duration of development [days] (SE;N)					
	Egg	1st instar larva	2nd instar larva	3rd instar larva	Pupa	Total development
14	9.63 (0.15; 8)	10.33 (0.23; 8)	10.31 (0.41; 8)	41.35 (0.07; 63)	18.19 (0.42; 54)	89.11 (0.45; 54)
15	6.55 (0.22; 8)	7.65 (0.17; 8)	7.15 (0.35 ;8)	33.89 (0.15; 319)	16.34 (0.12; 302)	71.29 (0.17; 302)
16	5.95 (0.12; 8)	5.27 (0.11; 8)	4.83 (0.11; 8)	28.97 (0.16; 373)	13.35 (0.08; 370)	57.78 (0.14; 370)
17	5.53 (0.08; 8)	9.54 (1.31; 8)	8.55 (0.52; 8)	26.90 (0.16; 240)	12.10 (0.10; 188)	60.98 (0.21; 188)
18	4.93 (0.15; 8)	4.13 (0.16; 8)	4.01 (0.16; 8)	23.97 (0.11; 354)	11.85 (0.10; 341)	48.81 (0.20; 341)
19	4.02 (0.12; 8)	4.04 (0.18; 8)	3.92 (0.21; 8)	22.61 (0.13; 380)	11.80 (0.06; 364)	45.14 (0.13; 364)
20	3.67 (0.06; 8)	3.96 (0.10; 8)	3.77 (0.15; 8)	22.27 (0.11; 400)	10.35 (0.07; 357)	43.75 (0.08; 357)
22	2.73 (0.09; 8)	2.35 (0.08; 8)	2.29 (0.08; 8)	13.46 (0.05; 390)	7.43 (0.02; 385)	28.28 (0.06; 385)
26	2.66 (0.04; 8)	1.55 (0.03; 8)	1.68 (0.08; 8)	12.82 (0.07; 338)	7.03 (0.06; 327)	25.65 (0.08; 327)
30	2.38 (0.09; 8)	1.39 (0.07; 8)	1.45 (0.06; 8)	11.89 (0.05; 187)	5.93 (0.07; 141)	22.84 (0.10; 141)

Immediately after hatching, the larvae had an average length of 6.91 ± 0.79 mm (Isomegalen diagram: Supplementary Fig. 1, Growth curves: Supplementary Figs. 2–10). At the growth peak, larvae were on average 25.14 ± 1.82 mm in length. Moulting usually occurred at a similar length of the larvae: 10.71 ± 0.79 mm for the first ecdysis and 16.10 ± 1.30 mm for the second ecdysis. Growth curves were distinctly sigmoidal (Supplementary Figs. 2–10).

**Figure 5:**
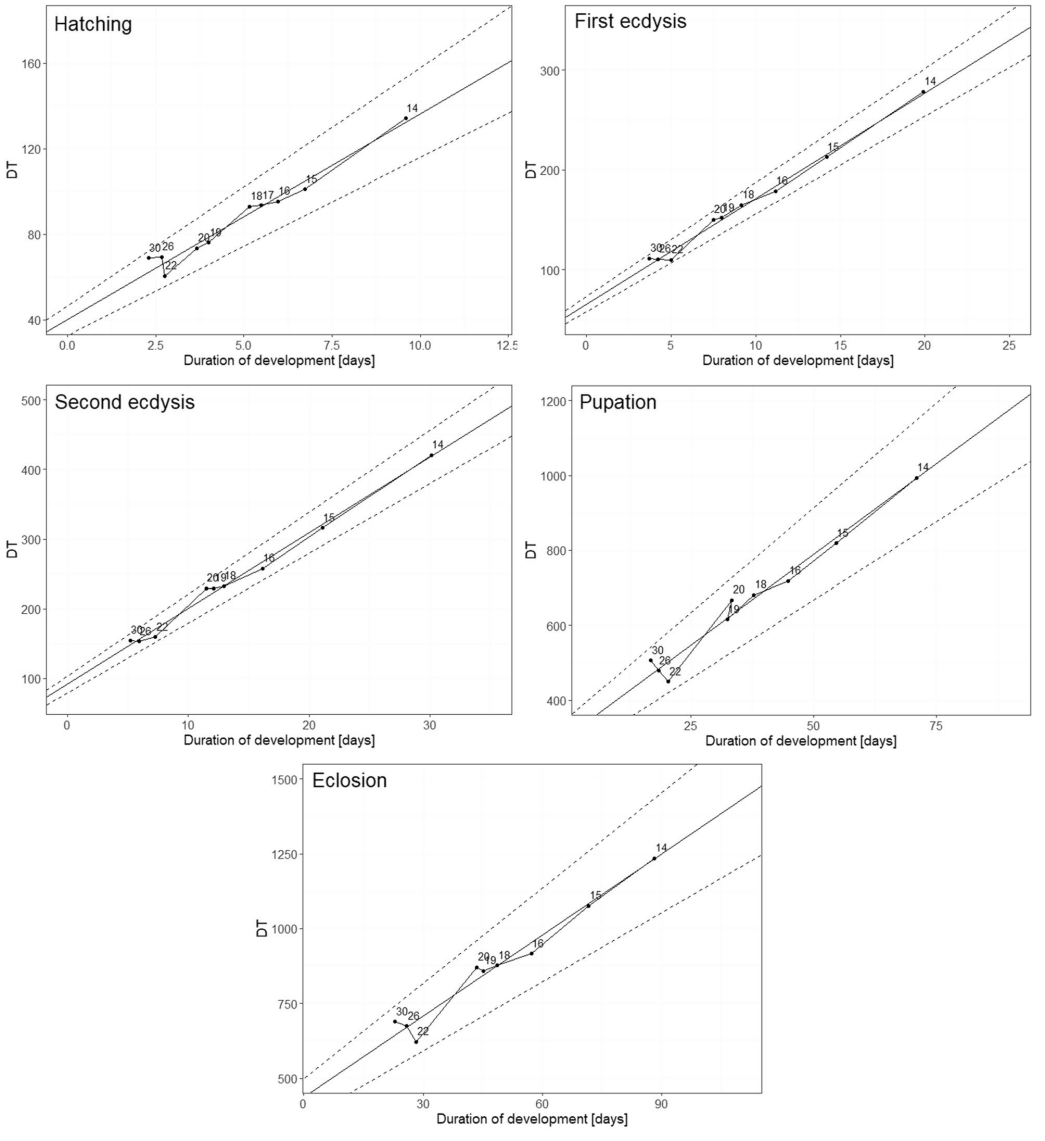
Reduced major axis (RMA) regression models for five developmental events of *N. littoralis.* DT is the time in days to reach developmental event multiplied by the constant rearing temperature. Temperature values are noted next to the points. Dashed lines represent 95% confidence intervals.

All included temperature points were within 95% confidence interval for all reduced major axis regression models (Fig. 5). The coefficient of determination for each thermal summation model exceeded 0.96, indicating high fit of the models. Lower developmental thresholds (*D*_*0*_) ranged from 9.04 °C for eclosion to 10.86 °C for second ecdysis. Thermal summation constants (*K*) were between 39.97 ADD for hatching and 434.705 ADD for eclosion (Table 3).

**Table 3:** Thermal summation models for five developmental events of *N. littoralis* calculated using Ikemoto and Takai method.

Developmental event	Temperature range [°C]	Model equation	Thermal summation constant—*K* (SE) [degree days]	Developmental threshold—*D*_*0*_ (SE) [°C]	N	r^2^	p
Hatching	14–30	*DT* = 39.977 + 9.627 × *D*	39.977 (2.902)	9.627 (0.539)	10	0.968	< 0.001
First ecdysis	14–30	*DT* = 65.747 + 10.583 × *D*	65.747 (3.235)	10.583 (0.315)	9	0.992	< 0.001
Second ecdysis	14–30	*DT* = 92.060 + 10.861 × *D*	92.060 (5.096)	10.861 (0.326)	9	0.992	< 0.001
Pupation	14–30	*DT* = 303.294 + 9.701 × *D*	303.294 (21.680)	9.701 (0.531)	9	0.973	< 0.001
Eclosion	14–30	*DT* = 434.705 + 9.044 × *D*	434.705 (28.862)	9.044 (0.548)	9	0.967	< 0.001

## Discussion

Current results on the development time are consistent with the development times of *N. littoralis* obtained by Dekeirsschieter^58^. She studied the development at two temperatures only. The development (without the egg stage) took on average 42.79 days at 18 °C and 23.60 days at 23 °C. By subtracting the egg stage from the mean development time at equivalent temperature, in the current study, we get consistent values. TSM for the eclosion from this paper is different compared to the general model for the total immature development given in our previous paper (Table 1 in Gruszka & Matuszewski^15^). The previous model was created without data from 17 °C and 22 °C, as regression analysis showed that they were outside the 95% confidence interval. In the current study, we excluded the data from 17 °C before the analysis and therefore they were not used while calculating initial TSMs (we give the reasons for this in the results section and discuss proximate and distant causes later in this section). Omitting data from 17 °C changed the results of the regression analysis, and the data point from 22 °C was now within the 95% confidence interval, so it was not omitted while deriving the current final TSMs. Therefore, current article gives more accurate thermal summation values for the eclosion of *N. littoralis* and we encourage using the current TSM for the eclosion instead of the less accurate previous one.

The value of thermal summation constant (*K*) for the eclosion of *N. littoralis* is larger compared to those obtained for other silphid beetles (Table 4). Larger accumulation of thermal units needed to reach maturity by an insect species indicates its longer development time. The development of *N. littoralis* is the longest among studied silphid beetles. Furthermore, this species usually colonizes cadavers later than for instance *Thanatophilus* species^59,60^. Therefore, *Necrodes littoralis* expands the timeframe in which PMI may be estimated using carrion beetles. In other beetle families, the thermal summation values are larger than in carrion beetles (Table 4).

**Table 4:** Comparison of thermal summation models for the eclosion created for forensically important beetles.

Species	Family	Geographic population	Temp. range [°C]	Thermal summation constant [ADD]	Developmental threshold [°C]	References
***Necrodes littoralis***	**Silphidae**	**Central European**	**14–30**	**434.70 ± 28.86**	**9.04 ± 0.55**	**This study**
*Thanatophilus micans*	Silphidae	South African	17–20	197.97 ± 19.74	13.26 ± 0.58	Ridgeway et al.^37^
*Thanatophilus mutilatus*	Silphidae	South African	15–27.5	384.11 ± 16.99	9.04 ± 0.36	Ridgeway et al.^37^
*Thanatophilus sinuatus*	Silphidae	Central European	14–26	360.46 ± 10.75	9.85 ± 0.23	Montoya-Molina et al.^39^
*Thanatophilus rugosus*	Silphidae	Central European	12–22	362.76 ± 4.97	8.53 ± 0.08	Montoya-Molina et al.^40^
*Dermestes tessellatocollis*	Dermestidae	Chinese	16–25	664.39 ± 55.87	12.07 ± 0.53	Wang et al.^42^
*Creophilus maxillosus*	Staphylinidae	Chinese	17.5–30	492.06 ± 23.61	9.60 ± 0.58	Wang et al.^44^
		Central European	15–30	405.16 ± 14.63	11.66 ± 0.24	Frątczak-Łagiewska et al.^45^
*Necrobia rufipes*	Cleridae	Chinese	22–36	591.00 ± 39.53	16.62 ± 0.63	Hu et al.^46^
*Omosita colon*	Nitidulidae	Chinese	16–31	514.1 ± 8.7	10.65 ± 0.16	Wang et al.^47^

The pattern of mortality across temperatures is consistent with the patterns revealed for other forensically important beetles. In *T.* *micans*, *T sinuatus*, *D. tessellatocollis*, *C. maxillosus* and *N. rufipes* the highest mortality was observed at extreme temperatures^35,39,42,45,46^. The same pattern is reported in this study. At intermediate temperatures, mortality of *N. littoralis* was below 25%, which is also in line with mortality patterns reported for the other beetle species^42,45^. In the study of Dekeirsschieter at 23 °C, mortality of *N. littoralis* was surprisingly high. It amounted to 30%^58^, whereas only 3.75% of the beetles died in 22 °C in the current study. Probably the higher mortality reported by Dekeirsschieter resulted from individual rearing of larvae, since mortality rate of individually reared larvae of *N littoralis* was found to be higher compared to the larvae that were reared in aggregations^61^. However, other effects might have been important, as well.

Mortality was exceptionally high at 17 °C in this study. More than half of the beetles died. Moreover, the development at this temperature deviated significantly from the general pattern, with the first and second instar larvae developing longer than at lower temperatures (16 °C and 15 °C). For this reason, we decided not to use data from 17 °C to calculate TSMs. Development extension and high mortality were probably caused here by rearing problems and were not directly related to the temperature itself. At 17 °C large numbers of nematodes were recorded in all containers. They were sticking to the larvae, which probably interrupted larval development and finally led to death of some larvae.

In order to create an isomegalen diagram and growth curves, it is necessary to measure larvae throughout their development. For this purpose, larvae can be killed and then measured. However, killing and storage methods may deform larvae^62^. Therefore, we decided to measure larvae in vivo. This method also has some drawbacks. First, the temperature changes when larvae are removed from the temperature chambers for measurement. Second, by taking a measurement, stress is induced that in turn can cause an increase in juvenile hormone levels, which interfere with the development^63^. Previous study^64^ revealed differences in development time between measured and non-measured *Creophilus maxillosus* beetles. However, after TSMs were built, it turned out that the estimation error did not differ significantly between the two models. The authors concluded that differences in development time were an effect of repeated stress rather than temperature changes during the measurements. Moreover, lack of significant differences between measured and non-measured TSMs was due to a small size of the differences and moderate size of the validation sample. In the current study, differences in the estimation error between the models were also insignificant. Based on this finding it can be concluded that in vivo measurements of forensically useful beetles have negligible impact on the resultant development models.

Third instar larvae in this study had longer maximum body length than those in previous studies of *N. littoralis*^58,65^. In the studies of Dekeirsschieter^58^, larvae were reared individually on small pieces of meat that could result in their smaller size. In the second study, larvae were collected from pig carcasses on the 17th, 20th and 24th days of decomposition^65^. The largest third instar larvae used in that study were therefore not the largest (longest) larvae present during decomposition, since after 24th day of decay larvae continued to feed and grow. In the current study, a substantial increase in larval length (above 22 mm) took place at the very end of the feeding phase (Supplementary Figs. 3–10). Fully mature Silphinae larvae range in size from 12 to 40 mm^66^, current results on larval length are therefore consistent with this range.

When rearing larvae in aggregations, it is difficult to ensure that manual samples are taken at random. We tried to sample larvae irrespective of their position inside the aggregation and their size. However owing to a natural tendency to pick larger larvae, there may be a size bias in our analysis.

A transition of the third instar larva to the post-feeding phase is a critical moment in *N. littoralis* development. This is when the larva buries itself into the soil to form a pupal chamber. The transition was impossible in the containers in which larvae were reared in aggregations. Therefore, it was necessary to transfer them to separate, smaller containers after they ceased feeding. This transfer might slightly interfere with development. Identification of the exact moment of transition to the post-feeding phase is, however, difficult due to rather long duration and lack of unambiguous markers of transition. The same problem was reported in the study of *D. tessellatocollis* development, in case of which durations of the last larval stage and the prepupal stage were summed up due to the difficulties in identifying the transition point^42^. In the study on *T. sinuatus, T. rugosu*s and *N. brunnicollis*, the post-feeding stage was distinguished^38–40^. Similarly, in the studies of *N. littoralis* by Dekeirsschieter, the post-feeding stage was identified, supposedly due to the rearing of larvae individually in Petri dishes that facilitated monitoring of the larvae^58^.

*Necrodes littoralis* is a species with great forensic potential. However, the lack of development models has largely limited its use in forensic cases. By providing the first comprehensive developmental dataset for this species, current study makes a significant contribution to the advancement of forensic entomology, particularly in central and northern Europe.

## Materials and methods

### Laboratory colony

Adult beetles came from our laboratory colony established in 2017 using insects collected in the alder forest of Biedrusko military range (Western Poland, Central Europe, 52° 31ʹ N, 16° 54ʹ E). They were kept at room temperature in medium-sized terrariums, 20–30 adult beetles per box (2–3 boxes maintained simultaneously). Boxes contained soil and cotton wool soaked with water. Beetles were fed with pork ad libitum.

### Experimental rearing

Laboratory rearing was conducted at ten constant temperatures: 14, 15, 16, 17, 18, 19, 20, 22, 26 and 30 °C. We followed the same protocol for each of the tested temperatures. Rearing was carried out inside the temperature chambers (type ST 1/1 BASIC or ST 1/ 1 + , POL-EKO, Poland). We controlled the chambers using temperature recorders. Deviations from the settings were consistent with the manufacturer's assurances (they did not exceed ± 0.5 °C). In order to induce oviposition adult beetles from the main colony were matched in pairs and placed in 0.5-L containers filled with soil. They had constant access to water and pork ad libitum. Containers were inspected every four hours for the presence of fresh eggs. If eggs were recorded, adult beetles were taken out from the container and eggs were left for hatching. Further inspections were carried at intervals representing no more than 10% of the egg stage duration. Inspection times were calculated based on the results of pilot studies. After hatching, first instar larvae were counted, and 50 larvae were placed in a small terrarium (18 cm × 11 cm × 14 cm) for further rearing. There were eight replicates per temperature (8 containers with 50 larvae each). Rearing boxes contained soil, cotton wool soaked with water, and pork meat ad libitum. Meat was covered with aluminum foil to avoid drying out. After feeding was completed and larvae started to bury themselves, they were transferred to new 0.5-L containers (8–10 larvae per container), filled with soil to allow them to form pupal chambers and complete their development.

### Inspections and measurements

Immature beetles from every terrarium were checked for transition to the next developmental stage: second instar larva, third instar larva, post-feeding larva, pupa and adult beetle. Inspections were carried out at time intervals that were no longer than 10% of the stage duration. In the case of the second instar, third instar and post-feeding larvae data were collected per container. Transitions were identified when more than half of the larvae passed to the next stage. Data for pupae and adult beetles were collected per insect. Mortality was measured for each stage of development. For this purpose, live individuals were counted after transition to the next stage. In addition, 24 sampled larvae from four out of eight containers (six larvae per container) were measured in vivo during each inspection. Length of their body was measured from the anterior margin of clypeus to the posterior end of the last abdominal segment. Measurements were made using a geometrical micrometer^67^. Larvae in the other four containers were inspected for transitions and mortality only.

### Data analysis

#### Influence of in vivo measurements

To test the influence of multiple in vivo measurements on the development of *N. littoralis*, we compared the total development time of measured and non-measured beetles. 100 randomly selected individuals were used per temperature (54 beetles at 14 °C due to high mortality). Non-parametric Mann–Whitney U test was used in this comparison. To account for the multiple tests we used Bonferonni correction (since there were 10 tests, we used 0.005 level of significance). Then, using data for measured and non-measured beetles, we created separate thermal summation models with the Ikemoto and Takai method^11^. In order to validate these models we used 110 non-measured beetles (15 beetles per temperature; insects from 17 °C were omitted; due to the high mortality, we had no specimens from 14 °C and only 5 beetles from 30 °C). The true physiological age of the insects (known based on laboratory rearing) was compared with the age estimated using the models. The differences between true and estimated age were used to quantify the estimation errors for measured and non-measured TSM. These errors were compared using the Wilcoxon signed-rank test. Analyzes were performed with Statistica 13 (TIBCO Software Inc.).

#### Mortality and development

We calculated the percentage mortality for each developmental stage and each of the temperatures tested. Due to the difficulty in accurate separation of the active feeding and post-feeding third instar larvae, we summed up the mortality for these stages. They were similarly combined for the purpose of all the subsequent analyzes. Using data for all individuals, we calculated the mean duration for each developmental stage at each temperature. Median times to reach developmental landmarks (i.e. hatching, first ecdysis, second ecdysis, pupation and eclosion) were calculated and used to build the isomorphen diagram. Using larval measurements, we built isomegalen diagram and growth curves. Thermal summation models were derived using the Ikemoto and Takai method^11^. For this purpose, we used measured and non-measured beetles. For hatching, first ecdysis and second ecdysis, the sample comprised eight observations per temperature, since we had eight containers for each temperature and could use only “container data” in these analyzes. For pupation and eclosion, the sample comprised 100 observations per temperature, since we could use “individual data” in these analyzes (for 14 °C the sample comprised 63 beetles upon pupation and 54 beetles upon eclosion). Growth curves and thermal summation models were created in R 3.5.2. The other analyzes were performed using Statistica 13 (TIBCO Software Inc.).

### Ethical approval

The study comprised laboratory experiments using insect species *Necrodes littoralis* (Coleoptera: Silphidae). The species is not under protection. No permission or approval from Ethic Commission were needed.

## Supplementary Information

Supplementary Information.

## Data Availability

The datasets generated and/or analyzed during the study are available from the corresponding author on a reasonable request.
