# Supplementary material for: Temperature models of development for Necrodes littoralis L. (Coleoptera: Silphidae), a carrion beetle of forensic importance in the Palearctic region
Source: Sci Rep. 2022 Jun 11;12:9689. doi: 10.1038/s41598-022-13901-y (PMC9188545; doi:10.1038/s41598-022-13901-y)
Supplement: Supplementary file 1 — Supplementary Information. [file 41598_2022_13901_MOESM1_ESM.pdf]

Supplementary information for

Temperature models of development for *Necrodes littoralis* L. (Coleoptera: Silphidae), a carrion beetle of forensic importance in the Palearctic region

Joanna Gruszka[1,2,3*], Szymon Matuszewski[1,2]

[1] Laboratory of Criminalistics, Adam Mickiewicz University, Św. Marcin 90, 61−809 Poznań, Poland
[2] Center for Advanced Technologies, Adam Mickiewicz University, Uniwersytetu Poznańskiego 10, 61−614 Poznań, Poland
[3] Department of Animal Taxonomy and Ecology, Adam Mickiewicz University, Uniwersytetu Poznańskiego 6, 61−614 Poznań, Poland
[*]corresponding author: joanna.gruszka@amu.edu.pl

Supplementary tables

Supplementary Table 1. Median time to reach developmental events for *N. littoralis* at nine constant temperatures. IQR – interquartile range

Temperature [°C]	Median time to reach a developmental event [days] (IQR)				
	Hatching	First ecdysis	Second ecdysis	Pupation	Eclosion
14	9.58 (0.58)	19.90 (0.71)	30.06 (2.69)	70.96 (1.13)	88.21 (3.75)
15	6.73 (1.02)	14.19 (1.31)	21.06 (1.65)	54.58 (3.96)	71.71 (3.29)
16	5.96 (0.44)	11.17 (0.29)	16.13 (0.58)	44.88 (5.35)	57.27 (4.13)
18	5.17 (0.69)	9.17 (1.10)	12.92 (1.00)	37.83 (3.17)	48.71 (3.17)
19	4.00 (0.67)	8.00 (0.33)	12.08 (1.17)	34.21 (3.75)	45.21 (3.83)
20	3.67 (0.33)	7.50 (0.33)	11.46 (0.63)	32.71 (2.04)	43.04 (1.79)
22	2.75 (0.27)	5.00 (0.29)	7.25 (0.08)	21.13 (1.33)	28.60 (1.69)
26	2.67 (0.13)	4.25 (0.13)	5.90 (0.33)	18.42 (1.13)	25.92 (1.77)
30	2.29 (0.33)	3.71 (0.42)	5.17 (0.50)	16.88 (1.00)	22.96 (1.42)

Supplementary figures

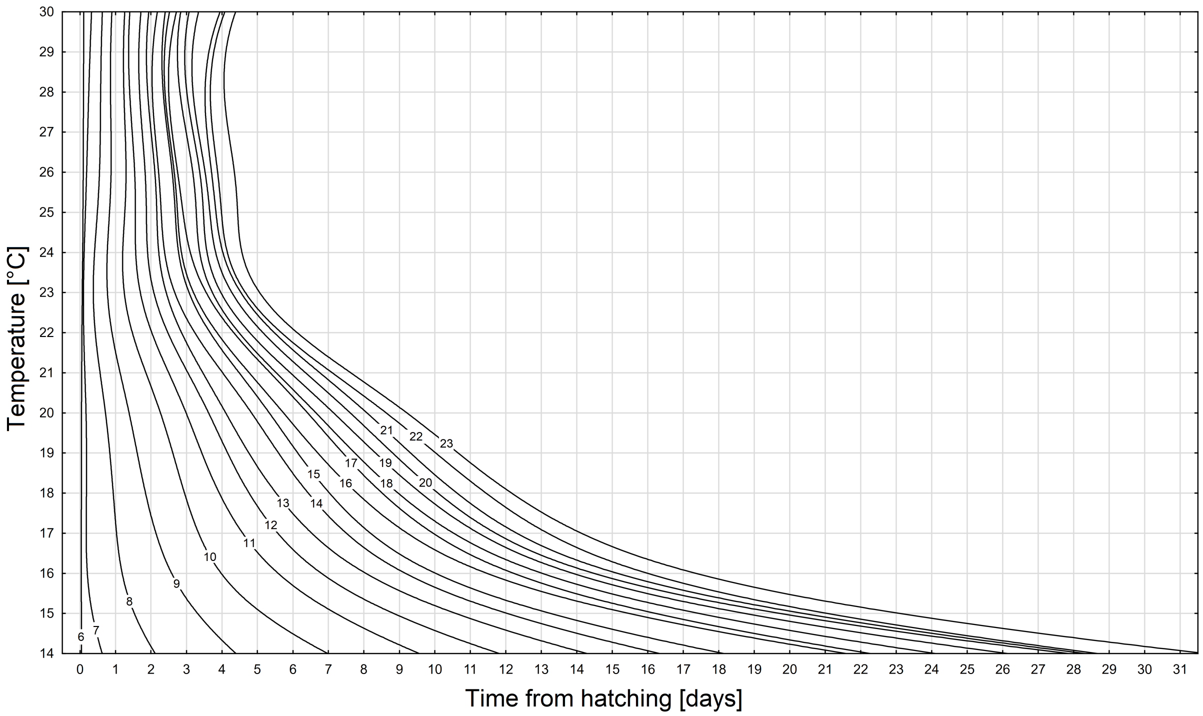

Supplementary Figure 1. Isomegalen diagram for *N. littoralis*. Each line represents larval body length.

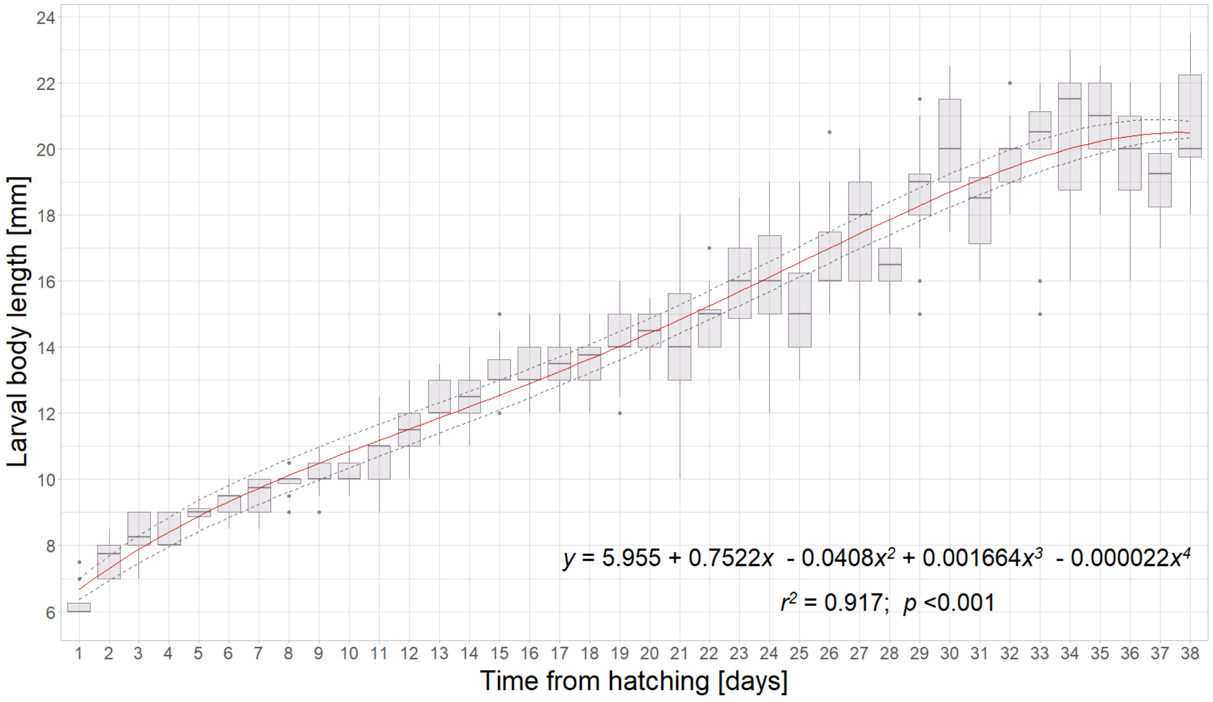

Supplementary Figure 2. Growth curve of *N. littoralis* larvae at 14°C. Boxplots show median, interquartile range, minimum and maximum body length. Dots are outliers. Red line represents polynomial model of the larval body length over the time of development in the active feeding phase (equation and statistics are given in the plot). Dashed lines represent standard error bounds.

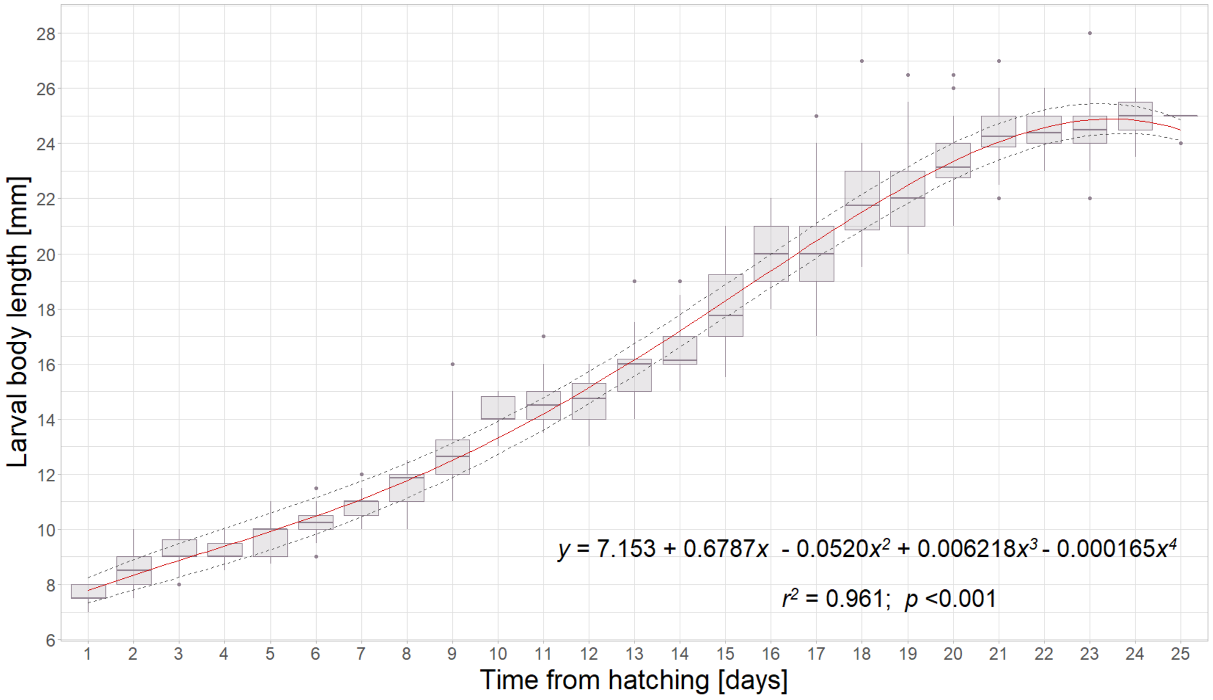

Supplementary Figure 3. Growth curve of *N. littoralis* larvae at 15°C. Boxplots show median, interquartile range, minimum and maximum body length. Dots are outliers. Red line represents polynomial model of the larval body length over the time of development in the active feeding phase (equation and statistics are given in the plot). Dashed lines represent standard error bounds.

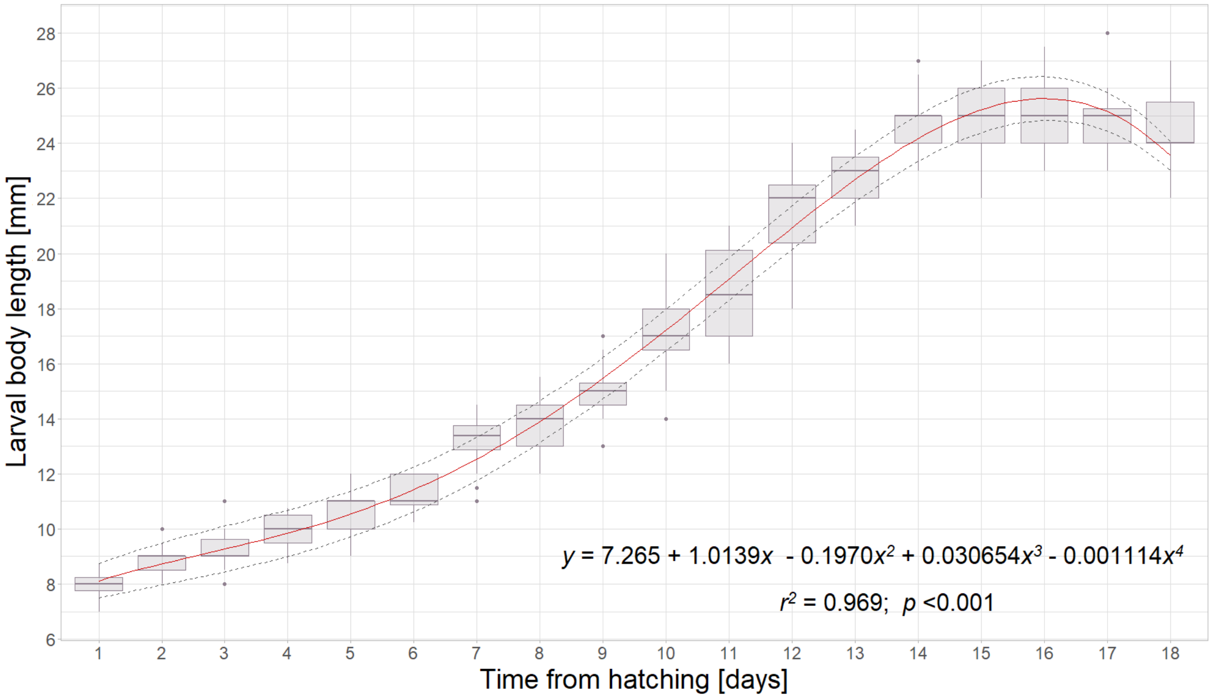

Supplementary Figure 4. Growth curve of *N. littoralis* larvae at 16°C. Boxplots show median, interquartile range, minimum and maximum body length. Dots are outliers. Red line represents polynomial model of the larval body length over the time of development in the active feeding phase (equation and statistics are given in the plot). Dashed lines represent standard error bounds.

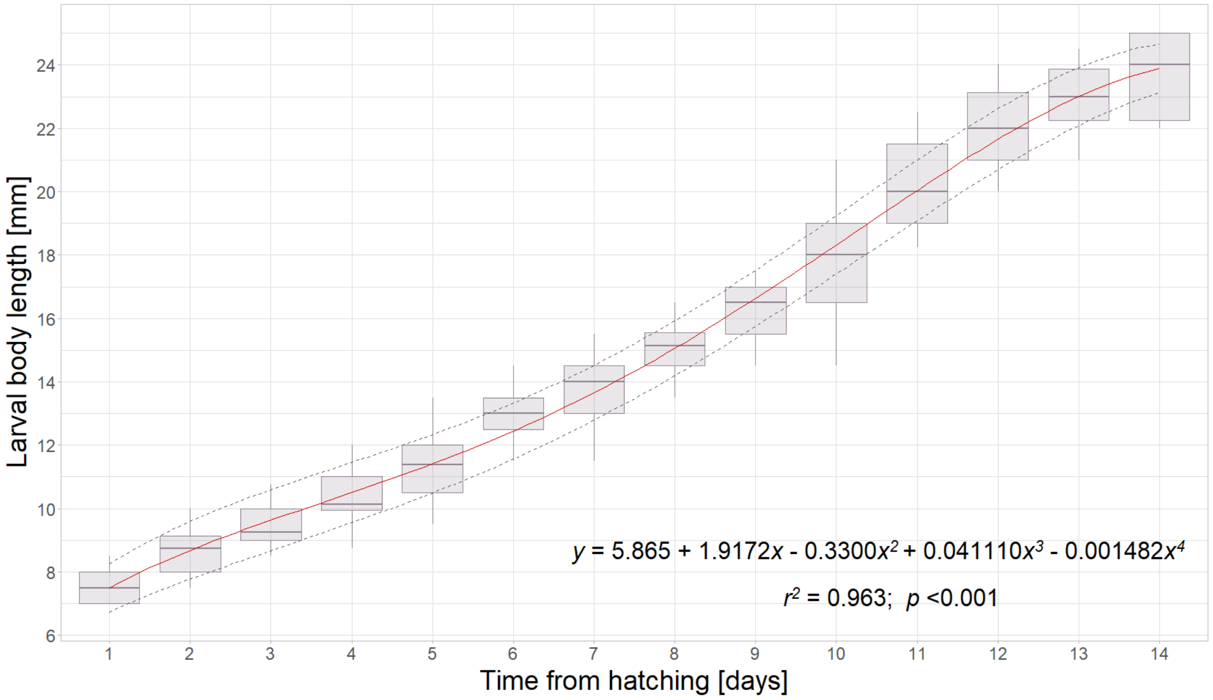

Supplementary Figure 5. Growth curve of *N. littoralis* larvae at 18°C. Boxplots show median, interquartile range, minimum and maximum body length. Red line represents polynomial model of the larval body length over the time of development in the active feeding phase (equation and statistics are given in the plot). Dashed lines represent standard error bounds.

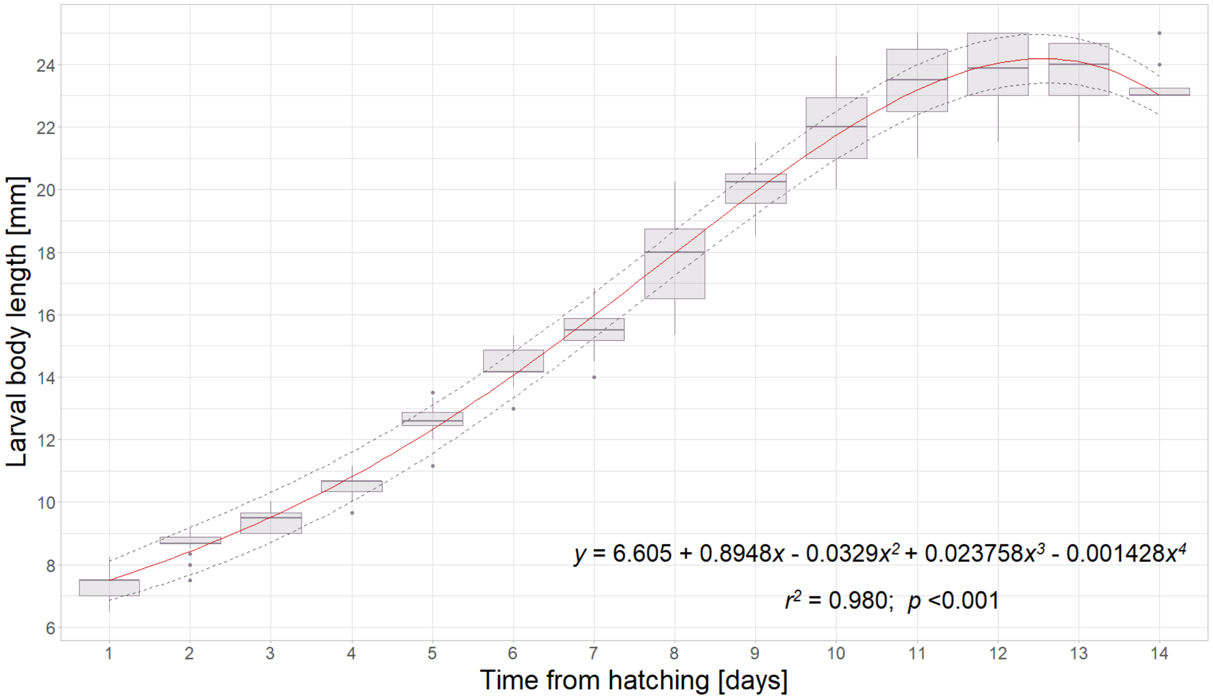

Supplementary Figure 6. Growth curve of *N. littoralis* larvae at 19°C. Boxplots show median, interquartile range, minimum and maximum body length. Dots are outliers. Red line represents polynomial model of the larval body length over the time of development in the active feeding phase (equation and statistics are given in the plot). Dashed lines represent standard error bounds.

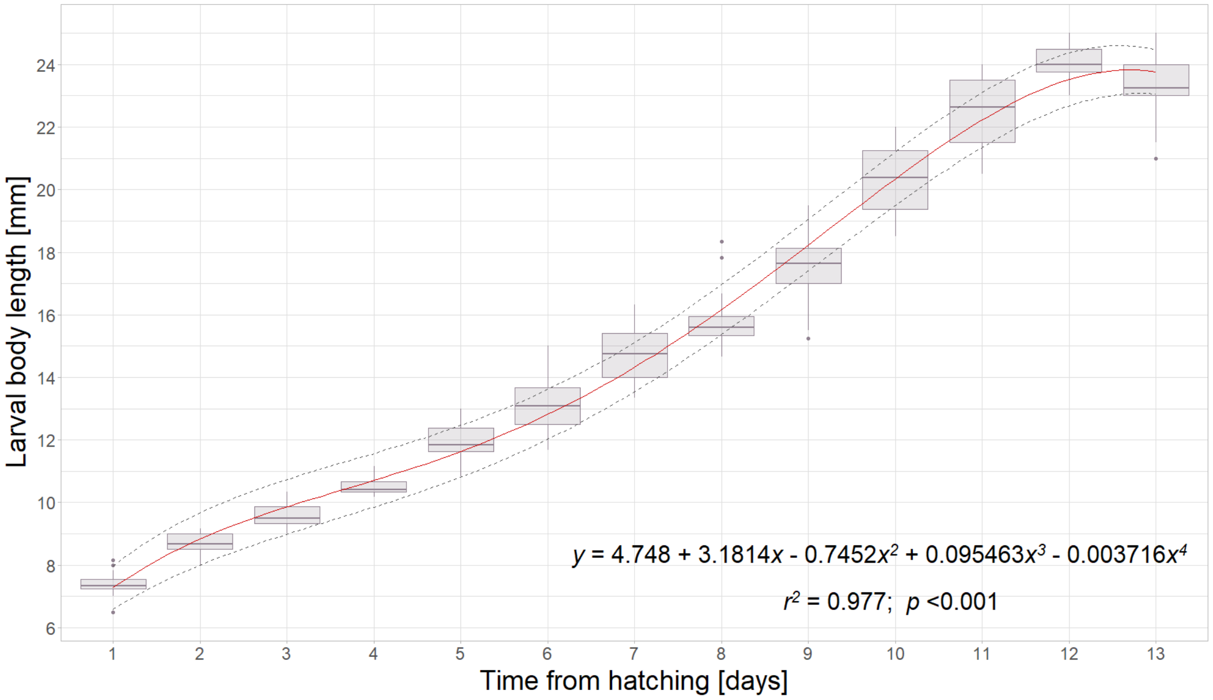

Supplementary Figure 7. Growth curve of *N. littoralis* larvae at 20°C. Boxplots show median, interquartile range, minimum and maximum body length. Dots are outliers. Red line represents polynomial model of the larval body length over the time of development in the active feeding phase (equation and statistics are given in the plot). Dashed lines represent standard error bounds.

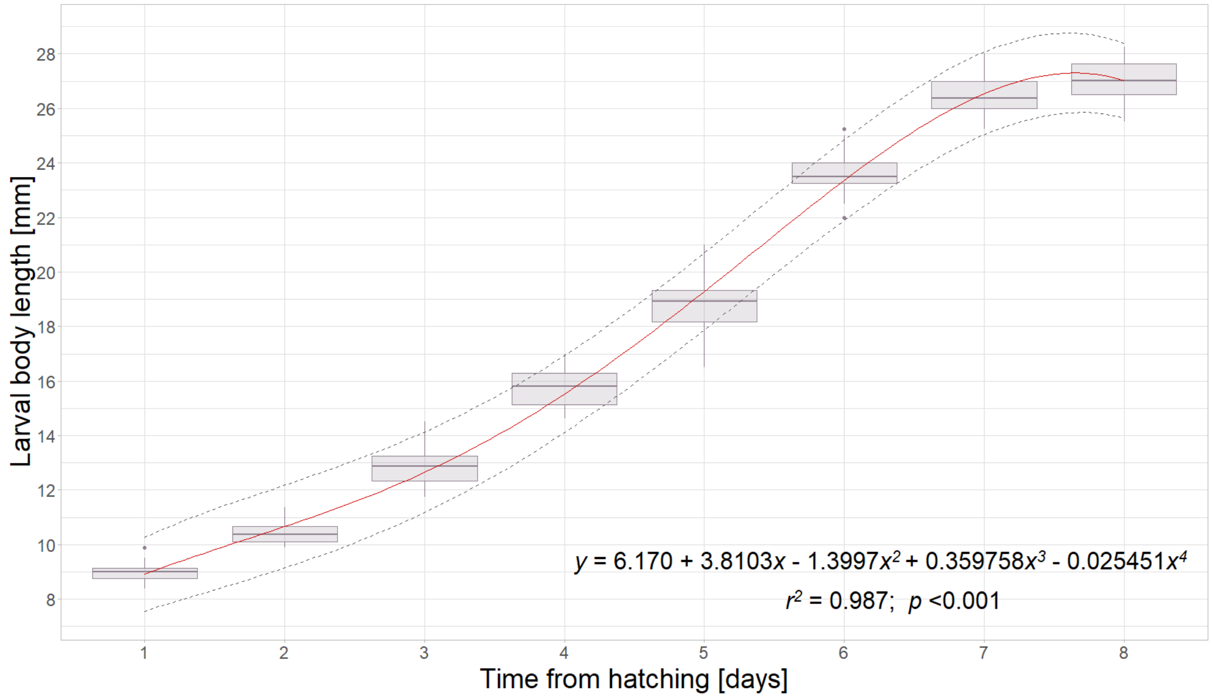

Supplementary Figure 8. Growth curve of *N. littoralis* larvae at 22°C. Boxplots show median, interquartile range, minimum and maximum body length. Dots are outliers. Red line represents polynomial model of the larval body length over the time of development in the active feeding phase (equation and statistics are given in the plot). Dashed lines represent standard error bounds.

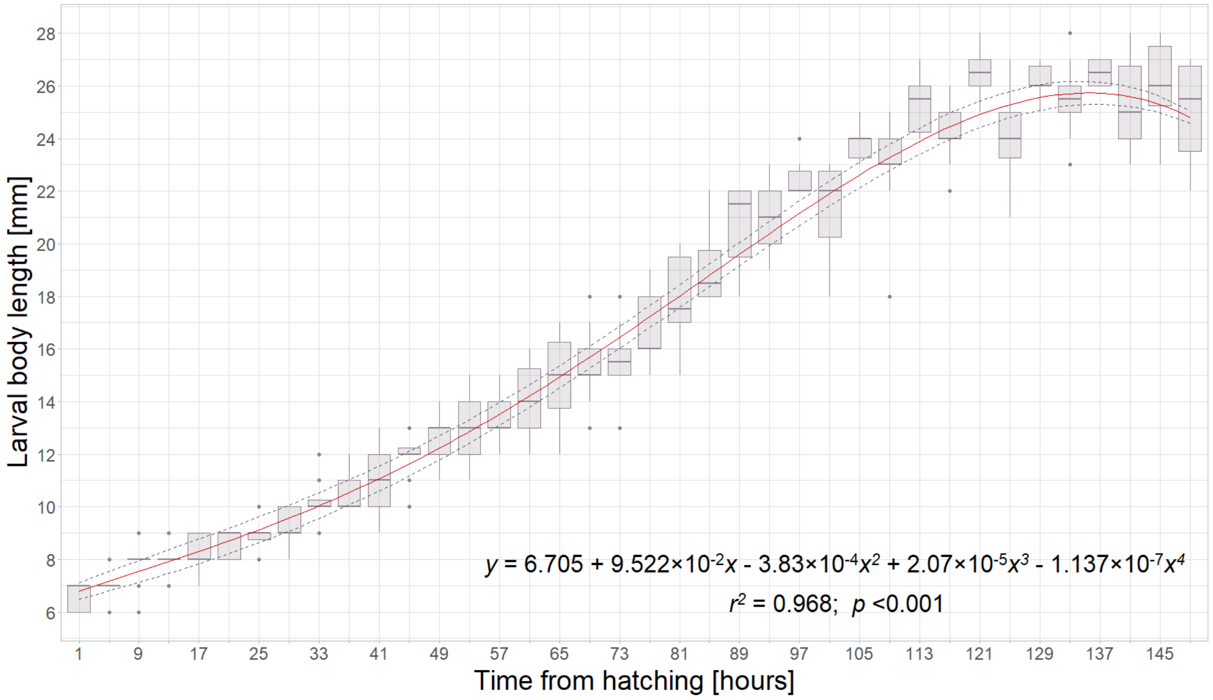

Supplementary Figure 9. Growth curve of *N. littoralis* larvae at 26°C. Boxplots show median, interquartile range, minimum and maximum body length. Dots are outliers. Red line represents polynomial model of the larval body length over the time of development in the active feeding phase (equation and statistics are given in the plot). Dashed lines represent standard error bounds.

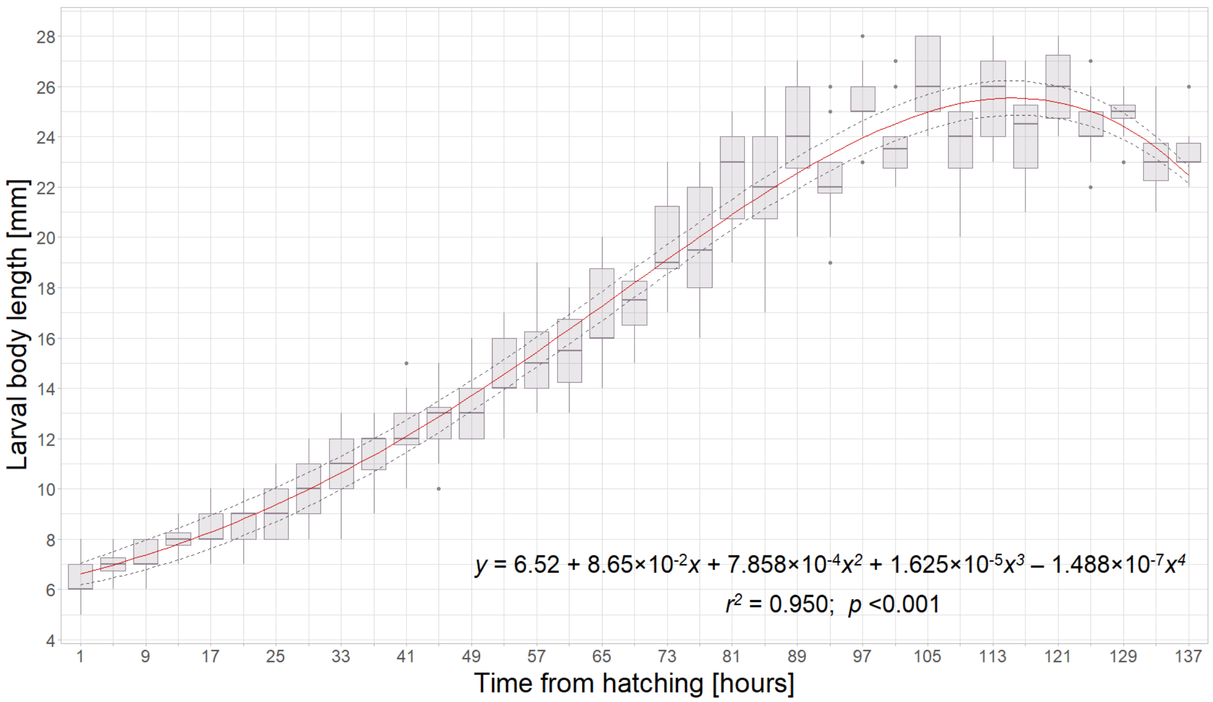

Supplementary Figure 10. Growth curve of *N. littoralis* larvae at 30°C. Boxplots show median, interquartile range, minimum and maximum body length. Dots are outliers. Red line represents polynomial model of the larval body length over the time of development in the active feeding phase (equation and statistics are given in the plot). Dashed lines represent standard error bounds.
